# Biomimetic construction of phospholipid membranes by direct aminolysis ligations

**DOI:** 10.1098/rsfs.2023.0019

**Published:** 2023-08-11

**Authors:** Federica A. Souto-Trinei, Roberto J. Brea, Neal K. Devaraj

**Affiliations:** ^1^ Biomimetic Membrane Chemistry (BioMemChem) Group, CICA—Centro Interdisciplinar de Química e Bioloxía, Universidade da Coruña, Rúa As Carballeiras, 15701 A Coruña, Spain; ^2^ Department of Chemistry and Biochemistry, University of California, San Diego, 9500 Gilman Drive, La Jolla, CA 92093, USA

**Keywords:** phospholipid, self-assembly, artificial cell, aminolysis, synthetic biology

## Abstract

Construction of artificial cells requires the development of straightforward methods for mimicking natural phospholipid membrane formation. Here we describe the use of direct aminolysis ligations to spontaneously generate biomimetic phospholipid membranes from water-soluble starting materials. Additionally, we explore the suitability of such biomimetic approaches for driving the *in situ* formation of native phospholipid membranes. Our studies suggest that non-enzymatic ligation reactions could have been important for the synthesis of phospholipid-like membranes during the origin of life, and might be harnessed as simplified methods to enable the generation of lipid compartments in artificial cells.

## 1. Background

The construction of artificial cells from purely synthetic components is an emerging area of bottom-up synthetic biology [1,2]. Artificial cells have the potential to shed light on numerous biological processes, as well as organize chemical reactions in specific compartments [3]. Therefore, there has been increasing interest in developing innovative strategies for the fabrication of synthetic cells. Chemoselective ligation chemistries have been demonstrated to be powerful tools for the convergent assembly of lipid fragments, providing straightforward methodologies for the construction of artificial phospholipid membranes [4]. One of the most efficient ligation strategies is native chemical ligation (NCL) [5,6]. While NCL is widely used for synthesizing large peptides and nucleic acids [7–9], this non-enzymatic and chemoselective approach has been also employed for rapidly preparing phospholipids *in situ* from water-soluble thioesters [10–13]. However, NCL is limited by the necessity for an *N*-terminal cysteine [5,6]. Therefore, cysteine-free ligation methodologies have also been developed for the synthesis of biomimetic phospholipids [14–18]. Rather than relying on *N*-terminal cysteines, we hypothesized that a simpler and straightforward method for phospholipid membrane generation could be built using direct aminolysis [19–23] as the key ligation reaction.

**Figure 1.**
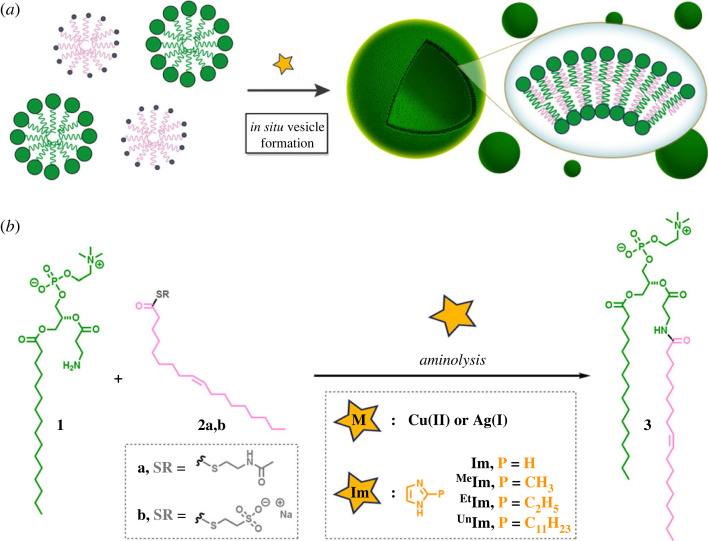
Biomimetic construction of phospholipid membranes based on direct aminolysis. (*a*) Schematic of spontaneous vesicle assembly induced by aminolysis-based amidophospholipid synthesis. (*b*) De novo formation of phospholipids (**3**) by metal- or imidazole-promoted aminolysis ligations between an amine-functionalized lysophospholipid (**1**) and an oleoyl thioester (**2a,b**).

Direct aminolysis uses the inherent nucleophilicity of *N*-terminal amines for the construction of amide bonds via reaction with *N*-acyl donors, notably, C-terminal thioesters [20–24]. These ligations tend to be significantly slower than NCL [25]. Reaction rates can be greatly increased by the addition of appropriate catalysts, such as metal ions or imidazole. Metal ion-assisted aminolysis has proven to be an effective methodology for the formation of relevant biomolecules [26–28]. For instance, the coordination of silver ions to peptide thioesters facilitates the acyl migration to amino moieties, which is frequently used for the preparation of cyclic peptides [29]. Alternatively, imidazole-catalysed aminolysis has been employed to synthesize large and complex peptides [30–32]. The mechanism of the reaction likely involves the reaction of imidazole with the C-terminal thioester, affording an acyl imidazole intermediate that subsequently reacts with the amine-containing ligation partner to form the final amide linkage. Considering the importance of this ligation strategy, it would be exciting to develop a straightforward methodology based on direct aminolysis to rapidly prepare phospholipids from water-soluble precursors. Here we describe the use of metal- and imidazole-promoted reactions for the *in situ* generation of phospholipid vesicles via direct aminolysis between amine-containing lysophospholipids and fatty acyl thioesters (figure 1).

## 2. Methods

### 2.1. Chemicals and materials

Commercially available 1-palmitoyl-2-hydroxy-*sn*-glycero-3-phosphocholine (**Lyso C_16_ PC**) and 1-palmitoyl-2-oleoyl-*sn*-glycerol-3-phosphocholine (**POPC**) were used as obtained from Avanti Polar Lipids. β-Alanine (H_2_N-β-Ala-OH), *N,N′*-diisopropylcarbodiimide (DIC), 4-dimethylaminopyridine (DMAP), trifluoroacetic acid (TFA), oleic acid, *N*-(3-dimethylaminopropyl)-*N′*-ethylcarbodiimide hydrochloride (EDC.HCl), *N*-acetyl cysteamine (SNAC), sodium 2-mercaptoethanesulfonate (MESNA) [33,34], copper(II) bromide (CuBr_2_), silver nitrate (AgNO_3_), tetrakis(acetonitrile)copper(I) hexafluorophosphate (Cu(CH_3_CN)_4_PF_6_), zinc iodide (ZnI_2_), manganese(II) bromide (MnBr_2_), nickel(II) iodide (NiI_2_), gold(I) chloride (AuCl), gold(III) chloride (AuCl_3_), silver(I) triflate (AgOTf), imidazole (Im), 1-methylimidazole (1-^Me^Im), 2-methylimidazole (2-^Me^Im), 4(5)-methylimidazole (4(5)-^Me^Im), 2-ethylimidazole (2-^Et^Im), 2-undecylimidazole (2-^Un^Im), 2-ethyl-4-methylimidazole (2-^Et^-4-^Me^Im), Boc-l-histidine (Boc-His-OH), histidine (H_2_N-His-OH), histamine, 1,6-diphenyl-1,3,5-hexatriene (DPH) and 8-hydroxypyrene-1,3,6-trisulfonic acid (HPTS) were obtained from Sigma-Aldrich. Texas Red 1,2-dihexadecanoyl-*sn*-glycero-3-phosphoethanolamine, triethylammonium salt (Texas Red DHPE) was obtained from Life Technologies. Deuterated chloroform (CDCl_3_), methanol (CD_3_OD) and DMSO (d_6_-DMSO) were obtained from Cambridge Isotope Laboratories. All reagents obtained from commercial suppliers were used without further purification unless otherwise noted. Analytical thin-layer chromatography was performed on E. Merck silica gel 60 F254 plates. Compounds, which were not UV active, were visualized by dipping the plates in a ninhydrin or potassium permanganate solution and heating. Silica gel flash chromatography was performed using E. Merck silica gel (type 60SDS, 230–400 mesh). Solvent mixtures for chromatography are reported as v/v ratios. HPLC analysis was carried out on an Eclipse Plus C8 analytical column with *Phase A*/*Phase B* gradients [*Phase A*: H_2_O with 0.1% formic acid; *Phase B*: MeOH with 0.1% formic acid]. HPLC purification was carried out on Zorbax SB-C18 semipreparative column with *Phase A*/*Phase B* gradients [*Phase A*: H_2_O with 0.1% formic acid; *Phase B*: MeOH with 0.1% formic acid]. Proton nuclear magnetic resonance (^1^H NMR) spectra were recorded on Varian VX-500 MHz or Jeol Delta ECA-500 MHz spectrometers and were referenced relative to residual proton resonances in CDCl_3_ (at 7.24 ppm), CD_3_OD (at 4.87 or 3.31 ppm) or d_6_-DMSO (at 2.50 ppm). Chemical shifts were reported in parts per million (ppm, *δ*) relative to tetramethylsilane (*δ* 0.00). ^1^H NMR splitting patterns are assigned as singlet (s), doublet (d), triplet (t), quartet (q) or pentuplet (p). All first-order splitting patterns were designated based on the appearance of the multiplet. Splitting patterns that could not be readily interpreted are designated as multiplet (m) or broad (br). Carbon nuclear magnetic resonance (^13^C NMR) spectra were recorded on Varian VX-500 MHz or Jeol Delta ECA-500 MHz spectrometers and were referenced relative to residual proton resonances in CDCl_3_ (at 77.23 ppm), CD_3_OD (at 49.15 ppm) or d_6_-DMSO (at 39.51 ppm). Electrospray ionization time of flight (ESI-TOF) spectra were obtained on an Agilent 6230 Accurate-Mass TOFMS mass spectrometer. Anisotropy measurements were obtained with a SPEX FluoroMax-3 spectrofluorometer. Transmission electron microscopy (TEM) images were recorded on an FEI TecnaiTM Sphera 200 kV microscope equipped with a LaB_6_ electron gun, using the standard cryotransfer holders developed by Gatan, Inc.

### 2.2. Synthesis of lysophospholipids

Lysophospholipid **1** was synthesized according to electronic supplementary material, scheme S1.

#### 2.2.1. *N*-Boc-β-Ala-OH (**4**)

To a solution of H_2_N-β-Ala-OH (500.0 mg, 5.61 mmol) in H_2_O (8.42 ml) were successively added Boc_2_O (8.42 ml, 8.42 mmol, 1 M solution in THF) and Et_3_N (2.35 ml, 16.83 mmol). After 3 h stirring at rt, the solution was acidified to pH 3 by the addition of HCl (10%), and extracted with CH_2_Cl_2_ (3 × 5 ml). The combined organic layers were dried (Na_2_SO_4_), filtered and concentrated, providing a yellow oil, which was purified by flash chromatography (0–3% MeOH in CH_2_Cl_2_), affording 928.0 mg of *N*-Boc-β-Ala-OH as a white solid (87%, R_f_ = 0.74 (70% MeOH in CH_2_Cl_2_)). ^1^H NMR (CDCl_3_, 500.13 MHz, *δ*): 6.28 (s, 0.3H, 0.3 × NH), 5.06 (s, 0.7H, 0.7 × NH), 3.40 (m, 2H, 1 × CH_2_), 2.58 (m, 2H, 1 × CH_2_), 1.44 (s, 9H, 3 × CH_3_). ^13^C NMR (CDCl_3_, 125.77 MHz, *δ*): 178.0 and 176.5, 157.8 and 156.1, 81.3 and 79.9, 37.3 and 36.0, 34.7, 28.6. MS (ESI-TOF) (*m/z* (%)): 212 ([M + Na]^+^, 100). HRMS (ESI-TOF) calculated for C_8_H_15_NO_4_Na ([M + Na]^+^) 212.0893, found 212.0895.

#### 2.2.2. 1-Palmitoyl-2-(*N*-Boc-β-Ala)-*sn*-glycero-3-phosphocholine (**5**)

A solution of *N*-Boc-β-Ala-OH (**4**, 61.1 mg, 322.6 µmol) in CH_2_Cl_2_ (15 ml) was stirred at rt for 10 min, and then DIC (93.8 µl, 604.8 µmol) and DMAP (24.6 mg, 201.6 µmol) were successively added. After 10 min stirring at rt, 1-palmitoyl-2-hydroxy-*sn*-glycero-3-phosphocholine (50.0 mg, 100.8 µmol) was added. After 12 h stirring at rt, the solvent was removed under reduced pressure, and the crude was purified by HPLC, affording 52.2 mg of **5** as a colourless foam (78%, t_R_ = 6.8 min (Zorbax SB-C18 semipreparative column, 5% *Phase A* in *Phase B*, 15.5 min)). ^1^H NMR (CDCl_3_, 500.13 MHz, *δ*): 6.15 (br s, 1H, 0.3H, 0.3 × NH), 5.41 (br s, 0.7H, 0.7 × NH), 5.21 (m, 1H, 1 × CH), 4.40–4.26 (m, 3H, 1.5 × CH_2_), 4.17–4.08 (m, 1H, 0.5 × CH_2_), 4.07–3.94 (m, 2H, 1 × CH_2_), 3.85–3.75 (m, 2H, 1 × CH_2_), 3.38–3–31 (m, 2H, 1 × CH_2_), 3.30 (s, 9H, 3 × CH_3_), 2.59–2.46 (m, 2H, 1 × CH_2_), 2.27 (t, *J* = 7.6 Hz, 2H, 1 × CH_2_), 1.61–1.50 (m, 2H, 1 × CH_2_), 1.40 (s, 9H, 3 × CH_3_), 1.30–1.20 (m, 24H, 12 × CH_2_), 0.86 (t, *J* = 6.8 Hz, 3H, 1 × CH_3_). ^13^C NMR (CDCl_3_, 125.77 MHz, *δ*): 173.8, 171.8, 156.1, 79.4, 70.9, 66.3, 64.2, 62.6, 59.8, 54.5, 36.4, 34.9, 34.2, 32.1, 29.9, 29.9, 29.8, 29.7, 29.5, 29.5, 29.3, 28.6, 25.0, 22.9, 14.3. MS (ESI-TOF) (*m/z* (%)): 689 ([M + Na]^+^, 100). HRMS (ESI-TOF) calculated for C_32_H_63_N_2_O_10_PNa ([M + Na]^+^) 689.4113, found 689.4114.

#### 2.2.3. 1-Palmitoyl-2-(β-Ala)-*sn*-glycero-3-phosphocholine (**1**)

A solution of 1-palmitoyl-2-(*N*-Boc-β-Ala)-*sn*-glycerol-3-phosphocholine (**5**, 9.0 mg, 13.5 µmol) in 1 ml of TFA/CH_2_Cl_2_ (1 : 1) was stirred at rt for 15 min. After the removal of the solvent, the residue was dried under high vacuum for 3 h. Then, the corresponding residue was diluted in MeOH (500 µl), filtered using a 0.2 µm syringe-driven filter, and the crude solution was purified by HPLC, affording 7.2 mg of the lysophospholipid **1** as a colourless oil (80%, t_R_ = 8.6 min (Zorbax SB-C18 semipreparative column, 50% *Phase A* in *Phase B*, 5 min, and then 5% *Phase A* in *Phase B*, 10 min)). ^1^H NMR (CD_3_OD, 500.13 MHz, *δ*): 5.19 (m, 1H, 1 × CH), 4.37–4.32 (dd, 1H, *J_1_* = 3.9 Hz, *J_2_* = 12.1 Hz, 0.5 × CH_2_), 4.26–4.16 (m, 3H, 1.5 × CH_2_), 4.14–4.06 (m, 1H, 0.5 × CH_2_), 4.03–3.95 (m, 1H, 0.5 × CH_2_), 3.61–3.56 (m, 2H, 1 × CH_2_), 3.22–3.13 (m, 2H, 1 × CH_2_), 3.17 (s, 9H, 3 × CH_3_), 2.82–2.65 (m, 2H, 1 × CH_2_), 2.28 (t, *J* = 7.4 Hz, 2H, 1 × CH_2_), 1.59–1.50 (m, 2H, 1 × CH_2_), 1.30–1.20 (m, 24H, 12 × CH_2_), 0.85 (t, *J* = 6.8 Hz, 3H, 1 × CH_3_). ^13^C NMR (CD_3_OD, 125.77 MHz, *δ*): 175.1, 171.5, 73.4, 67.5, 65.2, 63.3, 60.6, 54.8, 36.4, 34.9, 33.2, 32.8, 31.0, 31.0, 31.0, 31.0, 30.9, 30.9, 30.8, 30.7, 30.6, 30.4, 26.1, 23.9, 14.6. MS (ESI-TOF) (*m/z* (%)): 567 ([MH]^+^, 100). HRMS (ESI-TOF) calculated for C_27_H_56_N_2_O_8_P ([MH]^+^) 567.3769, found 567.3770.

### 2.3. Synthesis of thioesters

Thioesters **2a** and **2b** were synthesized according to electronic supplementary material, schemes S2*a* and S2*b*, respectively.

#### 2.3.1. SNAC oleoyl thioester (**2a**)

A solution of oleic acid (100.0 mg, 354.0 µmol) in CH_2_Cl_2_ (3.54 ml) was stirred at 0°C for 10 min, and then DMAP (4.3 mg, 35.4 µmol) and EDC.HCl (74.6 mg, 389.0 µmol) were successively added. After 10 min stirring at 0°C, SNAC (45.0 µl, 425.0 µmol) was added. After 5 h stirring at rt, the solvent was removed under reduced pressure, and the crude was purified by flash chromatography (10–50% EtOAc in hexanes), affording 131.1 mg of **2a** as a white solid (97%, R_f_ = 0.28 (50% EtOAc in hexanes)). ^1^H NMR (CDCl_3_, 500.13 MHz, *δ*): 6.08 (br s, 1H, 1 × NH), 5.35–5.23 (m, 2H, 2 × CH), 3.37 (q, *J* = 6.2 Hz, 2H, 1 × CH_2_), 2.97 (t, *J* = 6.5 Hz, 2H, 1 × CH_2_), 2.51 (t, *J* = 7.4 Hz, 2H, 1 × CH_2_), 1.98–1.92 (m, 4H, 2 × CH_2_), 1.92 (s, 3H, 1 × CH_3_), 1.65–1.54 (m, 2H, 1 × CH_2_), 1.32–1.16 (m, 20H, 10 × CH_2_), 0.83 (t, *J* = 6.8 Hz, 3H, 1 × CH_3_). ^13^C NMR (CDCl_3_, 125.77 MHz, *δ*): 200.2, 170.4, 130.1, 129.7, 44.2, 39.8, 32.0, 29.8, 29.7, 29.6, 29.4, 29.2, 29.1, 29.0, 28.5, 27.3, 27.2, 25.7, 23.3, 22.7, 14.2. MS (ESI-TOF) (*m/z* (%)): 406 ([M + Na]^+^, 100). HRMS (ESI-TOF) calculated for C_22_H_41_NO_2_SNa ([M + Na]^+^) 406.2750, found 406.2753.

#### 2.3.2. MESNA oleoyl thioester (**2b**)

A solution of oleic acid (189.2 mg, 670.0 µmol) in CH_2_Cl_2_ (5 ml) was stirred at 0°C for 10 min, and then DMAP (7.4 mg, 60.9 µmol) and EDC.HCl (128.4 mg, 670.0 µmol) were successively added. After 10 min stirring at 0°C, MESNA (100.0 mg, 609.1 µmol) was added. After 5 h stirring at rt, the mixture was extracted with H_2_O (2 × 3 ml) and the combined aqueous phases were washed with EtOAc (3 ml). After evaporation of H_2_O under reduced pressure, the residue was washed with CH_3_CN (5 ml) and then filtered to yield 194.7 mg of **2b** [44] as a white solid (75%). ^1^H NMR (d_6_-DMSO, 500.13 MHz, *δ*): 5.36–5.27 (m, 2H, 2 × CH), 3.05–2.99 (m, 2H, 1 × CH_2_), 2.60–2.51 (m, 4H, 2 × CH_2_), 2.02–1.92 (m, 4H, 2 × CH_2_), 1.58–1.49 (m, 2H, 1 × CH_2_), 1.34–1.18 (m, 20H, 10 × CH_2_), 0.85 (t, *J* = 6.9 Hz, 3H, 1 × CH_3_). ^13^C NMR (d_6_-DMSO, 125.77 MHz, *δ*): 198.7, 129.8, 129.7, 51.0, 43.4, 31.4, 29.2, 29.1, 28.9, 28.8, 28.7, 28.6, 28.5, 28.3, 26.7, 26.6, 25.1, 24.4, 22.2, 14.1. MS (ESI-TOF) (*m/z* (%)): 429 ([MH]^+^, 100). HRMS (ESI-TOF) calculated for C_20_H_38_NaO_4_S_2_ ([MH]^+^) 429.2104, found 429.2105.

### 2.4. Synthesis of phospholipids

Phospholipids **3** and **POPC** were synthesized according to electronic supplementary material, schemes S3*a* and S3*b*, respectively.

#### 2.4.1. Amidophospholipid 1-palmitoyl-2-[β-Ala-(oleoyl)]-*sn*-glycero-3-phosphocholine (**3**)

1-Palmitoyl-2-(β-Ala)-*sn-*glycerol-3-phosphocholine (**1**, 7.0 mg, 10.5 µmol) was treated with a 30 mM solution of MESNA oleoyl thioester (**2b**, 4.5 mg, 10.5 µmol) in 1.5 M solution of imidazole in H_2_O (351 µl), and the reaction was stirred and heated at 37°C. After 2 h, the mixture was diluted in MeOH (351 µl), filtered using a 0.2 µm syringe-driven filter, and the crude solution was purified by HPLC, affording 7.8 mg of the amidophospholipid **3** as a white solid (90%, t_R_ = 13.2 min (Zorbax SB-C18 semipreparative column, *Phase B*, 15.5 min)). ^1^H NMR (CDCl_3_, 500.13 MHz, *δ*): 6.92–6.82 (m, 1H, 1 × NH), 5.36–5.26 (m, 2H, 2 × CH), 5.24–5.16 (m, 1H, 1 × CH), 4.48–4.24 (m, 3H, 1.5 × CH_2_), 4.20–3.96 (m, 3H, 1.5 × CH_2_), 3.90–3.72 (m, 2H, 1 × CH_2_), 3.51–3.41 (m, 2H, 1 × CH_2_), 3.30 (s, 9H, 3 × CH_3_), 2.62–2.47 (m, 2H, 1 × CH_2_), 2.27 (t, *J* = 7.9 Hz, 2H, 1 × CH_2_), 2.14 (t, *J* = 7.5 Hz, 2H, 1 × CH_2_), 2.00–1.92 (m, 4H, 2 × CH_2_), 1.61–1.50 (m, 4H, 2 × CH_2_), 1.33–1.17 (m, 44H, 22 × CH_2_), 0.85 (t, *J* = 6.7 Hz, 6H, 2 × CH_3_). ^13^C NMR (CDCl_3_, 125.77 MHz, *δ*): 174.0, 173.9, 171.8, 130.2, 129.9, 76.8, 71.0, 66.5, 64.5, 62.4, 59.9, 54.7, 51.1, 36.7, 35.3, 34.6, 34.2, 32.2, 32.1, 30.0, 30.0, 29.9, 29.9, 29.9, 29.8, 29.6, 29.6, 29.5, 29.4, 29.3, 27.4, 27.4, 26.0, 25.1, 22.9, 22.9, 14.4. MS (ESI-TOF) (*m/z* (%)): 853 ([M + Na]^+^, 100). HRMS (ESI-TOF) calculated for C_45_H_87_N_2_O_9_PNa ([M + Na]^+^) 853.6041, found 853.6042.

#### 2.4.2. 1-Palmitoyl-2-oleoyl-*sn*-glycerol-3-phosphocholine

1-Palmitoyl-2-hydroxy-*sn*-glycero-3-phosphocholine (**Lyso C_16_ PC**, 5.2 mg, 10.5 µmol) was treated with a 30 mM solution of MESNA oleoyl thioester (**2b**, 4.5 mg, 10.5 µmol) in 1.5 M solution of imidazole in H_2_O (351 µl), and the reaction was stirred and heated at 37°C. After 2 h, the mixture was diluted in MeOH (351 µl), filtered using a 0.2 µm syringe-driven filter, and the crude solution was purified by HPLC, affording 6.9 mg of the phospholipid **POPC** as a white solid (86%, t_R_ = 14.1 min (Zorbax SB-C18 semipreparative column, *Phase B*, 15.5 min)). ^1^H NMR (CDCl_3_, 500.13 MHz, *δ*): 5.36–5.25 (m, 2H, 2 × CH), 5.20–5.09 (m, 1H, 1 × CH), 4.40–4.30 (m, 1H, 0.5 × CH_2_), 4.29–4.18 (m, 2H, 1 × CH_2_), 4.12–4.04 (dd, *J_1_* = 7.6 Hz, *J_2_* = 4.6 Hz, 1H, 0.5 × CH_2_), 3.99–3.80 (m, 2H, 1 × CH_2_), 3.73–3.65 (m, 2H, 1 × CH_2_), 3.28 (s, 9H, 3 × CH_3_), 2.31–2.20 (m, 4H, 2 × CH_2_), 2.03–1.91 (m, 4H, 2 × CH_2_), 1.62–1.47 (m, 4H, 2 × CH_2_), 1.36–1.14 (m, 44H, 22 × CH_2_), 0.85 (t, *J* = 6.7 Hz, 6H, 2 × CH_3_). ^13^C NMR (CDCl_3_, 125.77 MHz, *δ*): 173.8, 173.4, 130.2, 129.9, 70.6, 66.4, 63.6, 63.1, 59.5, 54.5, 34.5, 34.3, 32.1, 32.1, 30.0, 30.0, 30.0, 30.0, 29.9, 29.9, 29.8, 29.8, 29.8, 29.7, 29.6, 29.6, 29.5, 29.5, 29.5, 29.4, 29.4, 29.4, 27.4, 25.2, 25.1, 22.9, 14.4. MS (ESI-TOF) (*m/z* (%)): 760 ([M + H]^+^, 100). HRMS (ESI-TOF) calculated for C_42_H_83_NO_8_P ([M + H]^+^) 760.5851, found 760.5853.

### 2.5. Analysis of micelle size and critical micelle concentrations

In total, 100 µl of aqueous solutions (10 mM, 1 mM, 100 µM, 10 µM and 1 µM) of **2b** or **Lyso C_16_ PC** were analysed by dynamic light scattering (DLS) to determine the micelle sizes (electronic supplementary material, figure S2) and the critical micelle concentrations (CMCs) (electronic supplementary material, figure S3).

### 2.6. HPLC/ELSD/MS analysis

Phospholipid synthesis was performed in the appropriate conditions as described above. Aliquots of 1.5 µl of phospholipid (**3** or **POPC**) sample were taken at various time points, diluted with 50 µl of MeOH and analysed using an Eclipse Plus C8 analytical column (5% *Phase A* in *Phase B*, 5.5 min) with an evaporative light scattering detector (ELSD) at a flow of 1.0 ml min^−1^. For all LC/MS runs, solvent *Phase A* consisted of H_2_O with 0.1% formic acid and solvent *Phase B* of MeOH with 0.1% formic acid.

### 2.7. Fluorescence microscopy of phospholipid membrane vesicles

#### 2.7.1. Hydration method

In total, 10 µl of a 20 mM solution of phospholipid (**3** or **POPC**) in CHCl_3_ was added to a 1 ml vial, placed under N_2_ and dried for 15 min to prepare a lipid film. Then, 200 μl of H_2_O was added and the solution was tumbled at 25°C for 1 h. Afterwards, 0.1 µl of a 100 µM Texas Red DPHE dye solution in EtOH was added to 10 µl of this 1 mM aqueous solution of phospholipid (**3** or **POPC**) and the mixture was briefly agitated. The corresponding mixture was finally monitored by fluorescence and phase contrast microscopy to determine the vesicle structure.

#### 2.7.2. Sonication method

In total, 10 µl of a 20 mM solution of phospholipid (**3** or **POPC**) in CHCl_3_ was added to a 1 ml vial, placed under N_2_ and dried for 15 min to prepare a lipid film. Then, 200 µl of H_2_O was added, and the resulting mixture was sonicated with heat (≈55°C) for 1 h. Afterwards, 0.1 µl of a 100 µM Texas Red DPHE dye solution in EtOH was added to 10 µl of this 1 mM aqueous solution of phospholipid (**3** or **POPC**) and the mixture was briefly agitated. The corresponding mixture was finally monitored by fluorescence and phase contrast microscopy to determine the vesicle structure.

### 2.8. Transmission electron microscopy studies

#### 2.8.1. General

A deposition system (Balzers Med010) was used to evaporate a homogeneous layer of carbon. The samples were collected over 400 mesh Cu grids. The grids were then negatively stained with a solution of 1% (w/w) uranyl acetate. Micrographs were recorded on an FEI Tecnai Sphera microscope operating at 200 kV and equipped with a LaB_6_ electron gun, using the standard cryotransfer holders developed by Gatan, Inc. For image processing, micrographs were digitized in a Zeiss SCAI scanner with different sampling windows.

#### 2.8.2. Transmission electron microscopy measurements

Copper grids (formvar/carbon-coated, 400 mesh copper) were prepared by glow discharging the surface at 20 mA for 1.5 min. Once the surface for vesicle adhesion was ready, 3.5 µl of a 5 mM solution of phospholipid (**3** or **POPC**) in H_2_O (previously sonicated at ≈55°C for 1 h) was deposited on the grid surface. This solution was allowed to sit for 10 s before being washed away with 10 drops of glass distilled H_2_O and subsequent staining with 3 drops of 1% (w/w) uranyl acetate. The stain was allowed to sit for 10 s before wicking away with filter paper. All grid treatments and simple depositions were on the dark/shiny/glossy formvar-coated face of the grid (this side face up during glow discharge). Samples were then imaged via TEM, revealing the presence of several populations of spherical compartments (50–900 nm in diameter), consistent with a vesicle architecture.

### 2.9. Encapsulation experiments

#### 2.9.1. Encapsulation of 8-hydroxypyrene-1,3,4-trisulfonic acid (standard method)

In total, 10 µl of a 10 mM solution of phospholipid (**3** or **POPC**) in CHCl_3_ was added to a 1 ml vial, placed under N_2_ and dried for 15 min to prepare a lipid film. Then, 100 µl of 100 µM HPTS aqueous solution was added to the lipid film and briefly vortexed. The solution was tumbled at rt for 30 min. Afterwards, the resulting cloudy solution was diluted with an additional 200 µl of H_2_O and transferred to a 100 kDa molecular weight cut-off (MWCO) centrifugal membrane filter and centrifuged for 3 min at 10 000 rcf (Eppendorf 5415C). The solution was similarly washed an additional five times to remove any non-encapsulated dye. Then, 5 µl of the vesicle solution was placed on a clean glass slide, secured by a greased coverslip and imaged on a spinning disc confocal microscope (488 nm laser) to observe encapsulation of HPTS.

#### 2.9.2. Encapsulation of 8-hydroxypyrene-1,3,4-trisulfonic acid (inverse emulsion method)

In total, 60 µl of a 20 mM solution of phospholipid (**3** or **POPC**) in CHCl_3_ was added to a 1 ml vial, placed under N_2_ and dried for 15 min to prepare a lipid film. Then, 200 µl of mineral oil was added and placed under N_2_ to displace the air above the mineral oil. The resulting mixture was sonicated with heat (≈55°C) for 1 h. Afterwards, 100 µl of the amidophospholipid oil was added to a 1 ml Eppendorf tube. Then, 10 µl of the *upper buffer* (1 mM HPTS + 1 mM DTT + 50 mM NaCl + 200 mM sucrose in 100 mM HEPES buffer pH 7.5 solution) was added, and the resulting mixture was flicked and vortexed until it was a cloudy emulsion. The corresponding emulsion was added to a 1 ml Eppendorf tube containing 100 µl of the *lower buffer* (1 mM DTT + 50 mM NaCl + 200 mM glucose in 100 mM HEPES buffer pH 7.5 solution), so it floated on top. After waiting 10 min, the sample was centrifuged for 10 min at 9000–10 000 rcf. The sample was separated from the oil (either aspirating off the oil or using a syringe/needle to collect the sample from the bottom). The sample contained vesicles encapsulating HPTS which were then observed using fluorescence microscopy.

### 2.10. Anisotropy studies

#### 2.10.1. Vesicle preparation and extrusion

In total, 50 µl of a 20 mM solution of phospholipid (**3** or **POPC**) in CHCl_3_ was added to a 1 ml vial, placed under N_2_ and dried for 15 min to prepare a lipid film. The dried film was hydrated in 500 µl of H_2_O (final concentration of phospholipid: 2 mM). The solution was briefly vortexed and then sonicated with heat (approx. 55°C) for 45 min. Once the sample was fully hydrated, it was extruded through a 100 nm membrane.

#### 2.10.2. Anisotropy measurements

In total, 2 µl of a 500 µM solution of DPH in EtOH was added to 198 µl of 100 nm phospholipid (**3** or **POPC**) extruded vesicles (final concentration of DPH: 5 µM, 1% v/v). The solution was tumbled at 25°C overnight. Steady-state anisotropy was measured at different temperatures (from −10 to 40°C) on a Perkin spectrophotometer with a manual polarizer accessory and Peltier temperature controller.

## 3. Results and discussion

### 3.1. Synthesis of amphiphilic precursors

We initially synthesized two substrates to mimic the native precursors of the common phospholipid **POPC**: an amine-functionalized analogue of the lysophospholipid 1-palmitoyl-*sn*-glycerol-3-phosphocholine (**1**) (electronic supplementary material, scheme S1, figure S1) and an SNAC oleoyl thioester (**2a**) instead of oleoyl-CoA (electronic supplementary material, scheme S2, figure S1). It is worth noticing that **2a** is slightly soluble in water, and the addition of organic solvents assures the complete solubilization of **2a** and subsequent ligation. To facilitate direct aminolysis under exclusively aqueous conditions, we also synthesized a highly water-soluble thioester derivative, MESNA [33,34] oleoyl thioester (**2b**) (electronic supplementary material, scheme S2, figure S1) [13]. DLS experiments showed that precursor **2b** formed micelles of approximately 2.8 nm in diameter (electronic supplementary material, figure S2) with CMCs below 10 µM (electronic supplementary material, figure S3) [35].

### 3.2. Amidophospholipid **3** formation by directed aminolysis

We next explored the aminolysis reactions between the lysophospholipid **1** and the previously prepared oleoyl thioesters (**2a,b**) (figure 1). Amidophospholipid **3** formation was analysed over time using combined liquid chromatography (LC), mass spectrometry (MS) and evaporative light scattering detection (ELSD) measurements (electronic supplementary material, figure S1). Combination of the thioester (**2a,b**) and the lysophospholipid **1** in the appropriate conditions led to the production of **3** in aqueous conditions using millimolar concentrations of reactants (figure 1; electronic supplementary material, figure S1).

Synthesis of the amidophospholipid **3** by direct aminolysis between the amine-containing lysolipid **1** and the thioester **2a** was initially studied. As expected, in the absence of any catalyst, phospholipid formation was not observed in organic polar solvents both at rt (table 1, entry 1; electronic supplementary material, table S1, entry 1) and 37°C (electronic supplementary material, table S1, entry 2). Ligations in organic/aqueous mixtures (table 1, entry 2; electronic supplementary material, table S1, entry 3) or water without organic solvents (table 1, entries 3 and 4) showed identical results.

**Table 1.** Optimization of the direct aminolysis conditions for the synthesis of the amidophospholipid **3**.

entry	aminolysis**^a^**	thioester**^b^**	additive	solvent	*T* (°C)	*T* (h)	**3** (%)
1		**2a**		CH_3_CN	rt^c^	20	nd^d^
2		**2a**		CH_3_CN/H_2_O (1 : 1)	rt	20	nd
3		**2a**		H_2_O	rt	20	nd
4		**2a**		H_2_O	rt	37	nd
5	MIA	**2a**	CuBr_2_ (30 mM)	CH_3_CN/H_2_O (1 : 1)	rt	20	4
6	MIA	**2a**	CuBr_2_ (30 mM)	CH_3_CN/H_2_O (1 : 1)	37	20	11
7	MIA	**2a**	AgNO_3_ (30 mM)	CH_3_CN/H_2_O (1 : 1)	rt	20	23
8	MIA	**2a**	AgNO_3_ (30 mM)	CH_3_CN/H_2_O (1 : 1)	37	20	48
9	MIA	**2a**	CuBr_2_ (30 mM)	H_2_O	rt	20	nd
10	MIA	**2a**	CuBr_2_ (30 mM)	H_2_O	37	20	3
11	MIA	**2a**	AgNO_3_ (30 mM)	H_2_O	rt	20	6
12	MIA	**2a**	AgNO_3_ (30 mM)	H_2_O	37	20	21
13	IP	**2a**	Im (1.5 M)	H_2_O	rt	2	3
14	IP	**2a**	Im (1.5 M)	H_2_O	rt	20	65
15	IP	**2a**	Im (1.5 M)	H_2_O	37	2	8
16	IP	**2a**	Im (1.5 M)	H_2_O	37	20	81
17		**2b**		H_2_O	rt	20	nd
18		**2b**		H_2_O	37	20	nd
19	MIA	**2b**	CuBr_2_ (30 mM)	H_2_O	rt	20	6
20	MIA	**2b**	CuBr_2_ (30 mM)	H_2_O	37	20	14
21	MIA	**2b**	AgNO_3_ (30 mM)	H_2_O	rt	20	29
22	MIA	**2b**	AgNO_3_ (30 mM)	H_2_O	37	20	53
23	IP	**2b**	Im (1.5 M)	H_2_O	rt	2	88
24	IP	**2b**	Im (1.5 M)	H_2_O	rt	20	99
25	IP	**2b**	Im (1.5 M)	H_2_O	37	1	96
26	IP	**2b**	Im (1.5 M)	H_2_O	37	2	99
27	IP	**2b**	Im (1.5 M)	H_2_O	37	20	99
28	IP	**2b**	Boc-His-OH (1.5 M)	H_2_O	37	20	3
29	IP	**2b**	2-^Me^Im (1.5 M)	H_2_O	37	2	46
30	IP	**2b**	2-^Me^Im (1.5 M)	H_2_O	37	20	81
31	IP	**2b**	2-^Et^Im (1.5 M)	H_2_O	37	2	nd
32	IP	**2b**	2-^Et^Im (1.5 M)	H_2_O	37	20	2
33	IP	**2b**	2-^Un^Im (1.5 M)	H_2_O	37	2	3
34	IP	**2b**	2-^Un^Im (1.5 M)	H_2_O	37	20	4

^a^Metal ion-assisted (MIA) or imidazole-promoted (IP) aminolysis.

^b^Thioester (**2a,b**) concentration: 30 mM. Ratio 1 : 1 with the lysolipid **1** (30 mM).

^c^Room temperature (approx. 25°C).

^d^Not detected (nd).

Taking into consideration the slow rates of the aminolysis ligation, we next evaluated the generation of the phospholipid **3** in the presence of different additives. Metal ion-assisted aminolysis between **1** (30 mM) and **2a** (30 mM) using CuBr_2_ (30 mM) as additive in a mixture of CH_3_CN/H_2_O (1 : 1) led to the formation of the non-canonical phospholipid **3** in low conversion percentages (table 1, entries 5 and 6). Higher conversion rates were obtained by using AgNO_3_ as additive (table 1, entries 7 and 8). Use of copper(I) species (electronic supplementary material, table S1, entry 4) did not produce the desired phospholipid, demonstrating that copper(II)-based additives are more appropriate to accelerate aminolysis reactions. Phospholipid **3** synthesis was also not detected upon addition of divalent metal ions other than copper(II) (electronic supplementary material, table S1, entries 5–7) or gold species (electronic supplementary material, table S1, entries 8 and 9). Interestingly, decreased levels of phospholipid conversion were observed by using metal ion-assisted aminolysis approaches in water without organic solvent (table 1, entries 9–12). We believe that this effect is likely due to the low water solubility of the thioester **2a**.

Alternatively, imidazole-promoted aminolysis between lysolipid **1** and thioester **2a** resulted in higher conversion rates (table 1, entries 13–16), leading to good yields (81%) after 20 h at 37°C (table 1, entry 16).

To avoid the use of organic solvents and solubility issues related with the derivative **2a**, we next evaluated the aminolysis reaction between the lysophospholipid **1** and the water-soluble thioester **2b**. Preliminary experiments demonstrated that phospholipid **3** formation did not take place in the absence of additives (table 1, entries 17 and 18; electronic supplementary material, table S1, entries 10–13). Metal ion-assisted aminolysis afforded phospholipid **3** in low conversion percentages (table 1, entries 19 and 20) when CuBr_2_ was used. Better results were obtained when AgNO_3_ was employed (table 1, entries 21 and 22), observing conversion percentages higher than 50% (table 1, entry 22). Surprisingly, utilization of different silver(I) species rather than AgNO_3_ did not enable the generation of the desired phospholipid (electronic supplementary material, table S1, entries 14 and 15).

**Figure 2.**
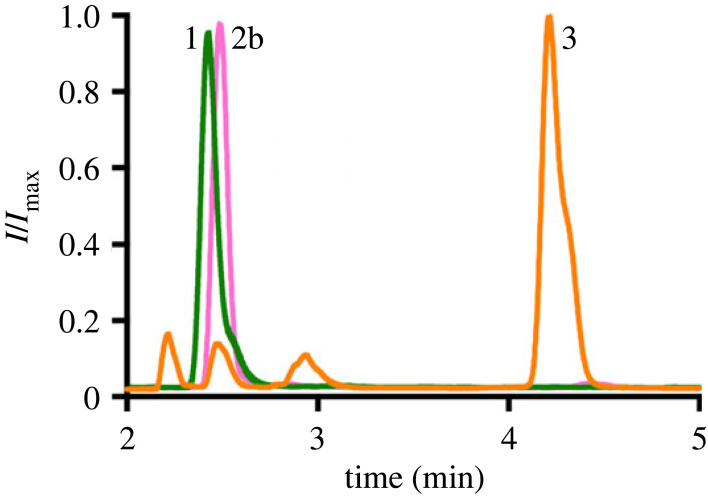
Monitoring amidophospholipid formation by HPLC/ELSD/MS. ELSD traces corresponding to the purified lysophospholipid **1**, MESNA oleoyl thioester **2b**, and amidophospholipid **3**. The retention times were verified by mass spectrometry and the use of known standards.

Imidazole-promoted aminolysis between **1** and **2b** resulted in higher conversion rates (table 1, entries 23–27) compared to the metal ion-assisted reactions. In the presence of imidazole, the addition of the thioester **2b** to the amine-containing lysophospholipid **1** immediately led to amidophospholipid **3** formation, and this process progressed to near completion after approximately 2 h using millimolar concentrations of reactants (figure 2; electronic supplementary material, figure S1). Interestingly, ligations in organic (electronic supplementary material, table S1, entry 16) or organic/aqueous mixtures (electronic supplementary material, table S1, entry 17) showed minimal phospholipid production. This is likely a consequence of the poor solubility of thioester **2b** in such organic solvents, thus hindering the ligation process.

Phospholipid **3** formation was only minimally observed when using Boc-His-OH, a relevant imidazole-containing amino acid derivative (table 1, entry 28; electronic supplementary material, table S1, entry 18). Taking this into consideration, we next studied the effect of the imidazole substitution on the aminolysis reaction. Interestingly, ligations in the presence of a monomethylated imidazole (2-^Me^Im) afforded phospholipid **3** conversion percentages from moderate (table 1, entry 29) to good (table 1, entry 30) yield depending on the analysed time scale. Alternatively, use of ethyl- (table 1, entries 31 and 32) or undecyl-based imidazoles (table 1, entries 33 and 34) resulted in much lower phospholipid conversions, probably as a consequence of a significant steric hindrance effect during the ligation process. These studies clearly demonstrated that non-substituted imidazole affords the highest conversion rates for the formation of the non-canonical amidophospholipid **3** via direct aminolysis in aqueous solution.

### 3.3. Anisotropy studies: determination of chain-melting temperatures in amidophospholipid **3**

**Figure 3.**
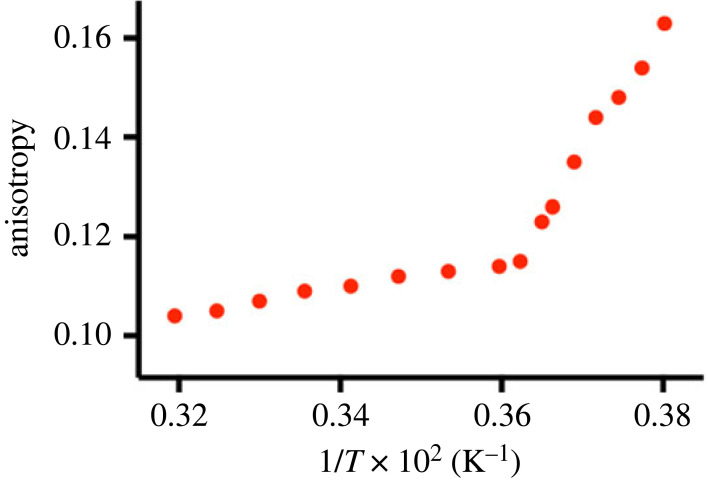
DPH anisotropy graph corresponding to a sample of phospholipid **3** vesicles. The melting temperature of the lipid chains in phospholipid **3** membranes was detected at 276 K.

We next determined the chain-melting temperature of the purified phospholipid **3** (figure 3). In particular, 1,6-diphenyl-1,3,5-hexatriene (DPH) anisotropy as a function of temperature was used to estimate the phase transition of the lipid chains [36]. A sudden change in the slope of the anisotropy indicates a transition from gel to liquid-crystalline phase (figure 3). The melting temperature of the lipid chains in **3** membranes was detected at 276 K. The measurements indicate that the amidophospholipid **3** membranes are well ordered, with fluidity and chain-melting temperatures comparable to those of native **POPC** membranes (*T_C_* = 270 K) [37].

### 3.4. Characterization of the amidophospholipid **3** vesicular structures

**Figure 4.**
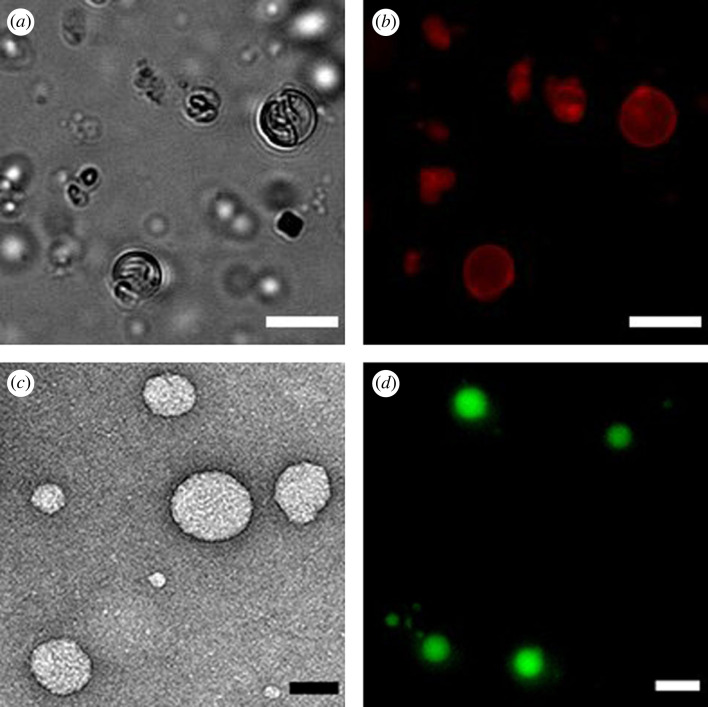
Characterization of the amidophospholipid vesicular structures. (*a*) Phase-contrast microscopy image of membrane vesicles resulting from the self-assembly of **3**. Scale bar denotes 5 µm. (*b*) Fluorescence microscopy image of vesicles formed by hydration of a thin film of **3**. Membranes were stained using 0.1 mol% Texas Red DHPE. Scale bar denotes 5 µm. (*c*) TEM image of negatively stained amidophospholipid **3** vesicles. Scale bar denotes 50 nm. (*d*) Fluorescence image demonstrating the encapsulation of HPTS in membrane vesicles of **3**. Scale bar denotes 5 µm.

We next performed microscopy studies to characterize the self-assembled lipid structures. As expected, neither the amine-modified lysophospholipid **1** nor the MESNA oleoyl thioester **2b** formed membranes in aqueous solution. However, the purified amidophospholipid **3**, when hydrated, readily formed membrane-bound vesicles. Lipid vesicular structures were initially identified by phase-contrast microscopy (figure 4*a*) and fluorescence microscopy using the membrane-staining dye Texas Red® DHPE (figure 4*b*). TEM also corroborated the formation of vesicular structures (figure 4*c*; electronic supplementary material, figure S4*a*). Additionally, amidophospholipid **3** vesicles were able to efficiently encapsulate polar fluorophore dyes, such as HPTS (figure 4*d*).

### 3.5. POPC formation by directed esterification

**Figure 5.**
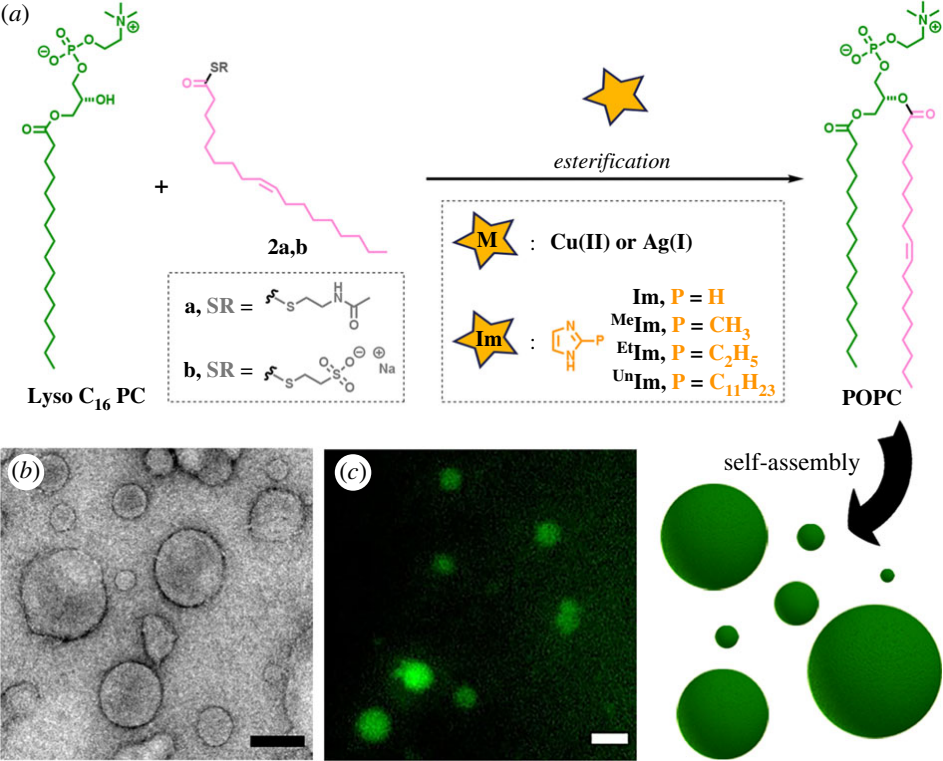
Construction of natural phospholipid membranes via direct esterification. (*a*) Synthesis of native phospholipids (**POPC**) by metal- or imidazole-promoted esterification ligations between a lysophospholipid (**Lyso C_16_ PC**) and an oleoyl thioester (**2a,b**). The corresponding lipid self-assembles into vesicular structures. (*b*) TEM image of negatively stained **POPC** liposomes. Scale bar denotes 100 nm. (*c*) Fluorescence image demonstrating the encapsulation of HPTS in **POPC** vesicles. Scale bar denotes 25 µm. Purified **POPC** employed for (*b*,*c*) was obtained via either metal ion-assisted or imidazole-promoted esterification.

Having shown that metal- and imidazole-promoted reactions enable the construction of biomimetic phospholipids by direct aminolysis ligations, we next evaluated if esterification ligations were feasible. Esterification between lysophospholipids and acyl thioesters would afford native phospholipids. Living organisms synthesize phospholipids through enzymatic acylation of lysophospholipids [38,39]. Nonetheless, enzyme-free esterification methods to form natural phospholipids in aqueous conditions remain both challenging and limited in scope [40,41]. Recent studies have shown successful monoacylation of lysophospholipids under alkaline conditions [42]. Considering the importance of developing a high-yielding synthesis of natural phospholipids in water [43], we explored the direct esterification between the lysophospholipid 1-palmitoyl-2-hydroxy-*sn*-glycero-3-phosphocholine (**Lyso C_18_ PC**) and the previously prepared oleoyl thioesters (**2a,b**) (figure 5). **POPC** synthesis was analysed over time using HPLC-ELSD-MS (electronic supplementary material, figure S1). The addition of the thioester (**2a,b**) to the lysophospholipid **Lyso C_16_ PC** in the appropriate conditions led to **POPC** formation in aqueous conditions using millimolar concentrations of reactants (figure 5; electronic supplementary material, figure S1).

We initially analysed the formation of **POPC** by direct esterification between **Lyso C_16_ PC** and the thioester **2a**. As expected, in the absence of any catalyst, phospholipid formation was not observed (table 2, entries 1 and 2; electronic supplementary material, table S2, entry 1). Surprisingly, addition of metals or imidazole did not enable an efficient synthesis of the corresponding natural phospholipid (table 2, entries 3–5; electronic supplementary material, table S2, entries 2 and 3), highlighting the complexity of such esterification reactions in aqueous conditions.

**Table 2.** *.* Optimization of the esterification conditions for the synthesis of **POPC**.

entry	esterification**^a^**	thioester**^b^**	additive	solvent	*T* (°C)	*T* (h)	**POPC** (%)
1		**2a**		CH_3_CN/H_2_O (1 : 1)	rt^c^	20	nd^d^
2		**2a**		CH_3_CN/H_2_O (1 : 1)	37	20	nd
3	MIA	**2a**	CuBr_2_ (30 mM)	CH_3_CN/H_2_O (1 : 1)	rt	20	2
4	MIA	**2a**	AgNO_3_ (30 mM)	CH_3_CN/H_2_O (1 : 1)	rt	20	3
5	IP	**2a**	Im (1.5 M)	H_2_O	rt	20	5
6		**2b**		H_2_O	rt	20	nd
7		**2b**		H_2_O	37	20	nd
8	MIA	**2b**	CuBr_2_ (30 mM)	H_2_O	rt	20	3
9	MIA	**2b**	AgNO_3_ (30 mM)	H_2_O	rt	20	4
10	IP	**2b**	Im (1.5 M)	H_2_O	rt	2	6
11	IP	**2b**	Im (1.5 M)	H_2_O	rt	20	21
12	IP	**2b**	Im (1.5 M)	H_2_O	37	2	15
13	IP	**2b**	Im (1.5 M)	H_2_O	37	20	54
14	IP	**2b**	Im (1.5 M) + CuBr_2_ (30 mM)	H_2_O	37	2	9
15	IP	**2b**	Im (1.5 M) + AgNO_3_ (30 mM)	H_2_O	37	2	15
16	IP	**2b**	1-^Me^Im (1.5 M)	H_2_O	rt	20	nd
17	IP	**2b**	1-^Me^Im (1.5 M)	H_2_O	37	20	5
18	IP	**2b**	2-^Me^Im (1.5 M)	H_2_O	rt	20	5
19	IP	**2b**	2-^Me^Im (1.5 M)	H_2_O	37	20	7
20	IP	**2b**	4(5)-^Me^Im (1.5 M)	H_2_O	rt	20	9
21	IP	**2b**	4(5)-^Me^Im (1.5 M)	H_2_O	37	20	12
22	IP	**2b**	2-^Et^Im (1.5 M)	H_2_O	rt	20	4
23	IP	**2b**	2-^Et^Im (1.5 M)	H_2_O	37	20	5
24	IP	**2b**	2-^Un^Im (1.5 M)	H_2_O	rt	20	3
25	IP	**2b**	2-^Un^Im (1.5 M)	H_2_O	37	20	4
26	IP	**2b**	2-^Et^-4-^Me^Im (1.5 M)	H_2_O	rt	20	2
27	IP	**2b**	2-^Et^-4-^Me^Im (1.5 M)	H_2_O	37	20	3

^a^Metal ion-assisted (MIA) or imidazole-promoted (IP) esterification.

^b^Thioester (**2a,b**) concentration: 30 mM. Ratio 1 : 1 with the **Lyso C_16_ PC** (30 mM).

^c^Room temperature (approx. 25°C).

^d^Not detected (nd).

Expecting an improved reactivity by using more water-soluble precursors, we next studied the esterification reaction between **Lyso C_16_ PC** and the thioester **2b**. Initial experiments showed no formation of **POPC** in the absence of catalysts (table 2, entries 6 and 7). Metal ion-assisted esterifications led to the formation of the natural phospholipid in very low conversions (table 2, entries 8 and 9). Alternatively, imidazole-promoted esterifications resulted in higher conversion rates (table 2, entries 10–13), with the best conditions leading to moderate conversion (54%) after 20 h at 37°C (table 2, entry 13). While modest, these ligation yields are promising, especially if we consider that these reactions result in the synthesis of natural phospholipids in aqueous solution by a one-pot enzyme-free esterification method. Addition of metals to the imidazole medium did not lead to an increase in the conversion rates of the esterification reaction (table 2, entries 14 and 15).

Interestingly, **POPC** formation was not observed using biologically relevant imidazole-containing molecules, such as histidine or histamine (electronic supplementary material, table S2, entries 4–9). We thus believe that the imidazole substitution and its hydrophobic character significantly affect the phospholipid ligation. Taking this into consideration, we evaluated the formation of **POPC** by direct esterification reactions in the presence of different alkylimidazoles (table 2, entries 16–27). In the case of monomethylated imidazoles, we observed that derivatives with a methyl group in the position 4(5) (table 2, entries 20 and 21) led to higher **POPC** conversions than imidazoles with methyl substitutions in the positions 1 (table 2, entries 16 and 17) or 2 (table 2, entries 18 and 19). Regarding the hydrophobic character, we observed that incorporation of larger alkyl substitutions (i.e. ethyl, undecyl) (table 2, entries 22–25) resulted in lower conversions, likely due a steric effect during the ligation process. Double-substituted imidazoles also resulted in very low conversions (table 2, entries 26 and 27), probably due to a similar effect. These results showed that non-substituted imidazole offers the highest conversion rates for the synthesis of natural **POPC**.

### 3.6. Anisotropy studies: determination of chain-melting temperatures in **POPC**

DPH anisotropy measurements on purified **POPC** samples obtained via either metal ion-assisted or imidazole-promoted esterification showed that the melting temperature of the lipid chains was 270 K, which corresponds to the values previously reported in the literature for **POPC** membranes (electronic supplementary material, figure S5) [37].

### 3.7. Characterization of the **POPC** vesicular structures

A thin film of the purified **POPC**, when hydrated, readily formed micrometre-sized vesicles (figure 5; electronic supplementary material, figure S6). Vesicular assemblies were identified by phase-contrast microscopy (electronic supplementary material, figure S6*a*) and fluorescence microscopy using Texas Red DHPE (electronic supplementary material, figure S6*b*). Confirmation that the resulting structures were membrane compartments was also achieved by negative staining TEM (figure 5*b*; electronic supplementary material, figure S4*b*). We also demonstrated that the corresponding liposomes were capable of encapsulating HPTS (figure 5*c*).

## 4. Conclusion

Here we report that direct aminolysis ligations allow the preparation of a new class of amidophospholipids, which spontaneously self-assemble to form vesicular structures. Thus, aminolysis reactions can be efficiently used as a non-enzymatic methodology to drive the de novo self-assembly of amidophospholipid membranes. Such biomimetic membranes could be employed in applications involving synthetic cells, as well as in the construction of drug-delivery systems and microreactors. Additionally, we have explored the suitability of the non-enzymatic approaches for driving the *in situ* formation of native phospholipid membranes via direct esterification. These studies could bring new insights into the origin of cellular membranes and open up multiple avenues for the straightforward non-enzymatic synthesis of membrane-forming phospholipids for artificial cells.

## Data Availability

The data are provided in electronic supplementary material [44].
